# Does Consulting an Occupational Medicine Specialist Decrease Time to Return to Work Among Total Knee Arthroplasty Patients? A 12-Month Prospective Multicenter Cohort Study

**DOI:** 10.1007/s10926-022-10068-1

**Published:** 2022-09-09

**Authors:** Y. van Zaanen, A. J. Kievit, R. C. I. van Geenen, T. M. J. Pahlplatz, M. J. M. Hoozemans, L. Blankevoort, M. U. Schafroth, D. Haverkamp, T. M. J. S. Vervest, D. H. P. W. Das, V. A. Scholtes, A. J. van der Beek, P. P. F. M. Kuijer

**Affiliations:** 1 Department of Public and Occupational Health, Amsterdam Public Health Research Institute, Amsterdam Movement Sciences, Amsterdam UMC, University of Amsterdam, Location AMC, Meibergdreef 9, 1105 AZ Amsterdam, Netherlands; 2 Orthopaedic Research Center Amsterdam, Amsterdam UMC, University of Amsterdam, Amsterdam, Netherlands; 3 Department of Orthopaedic Surgery, Amphia Hospital, Breda, Netherlands; 4 Department of Human Movement Sciences, Vrije Universiteit Amsterdam, Amsterdam, Netherlands; 5 Xpert Orthopaedics, Amsterdam, Netherlands; 6 Department of Orthopaedic Surgery, Tergooi Hospital, Hilversum, Netherlands; 7 Department and Research Center of Orthopaedic Surgery, St. Anna Hospital, Geldrop, Netherlands; 8 Joint Research Orthopedic Surgery, OLVG+, Amsterdam, Netherlands; 9 Department of Public and Occupational Health, Amsterdam Public Health Research Institute, Amsterdam UMC, Vrije Universiteit Amsterdam, Amsterdam, Netherlands

**Keywords:** Total knee arthroplasty (TKA), Return to work (RTW), Occupational medicine specialist (OMS), Expectations

## Abstract

**Purpose:**

The aim of this study is to investigate whether total knee arthroplasty (TKA) patients who consulted an occupational medicine specialist (OMS) within 3 months after surgery, return to work (RTW) earlier than patients who did not consult an OMS.

**Methods:**

A multi-center prospective cohort study was performed among working TKA patients, aged 18 to 65 years and intending to RTW. Time to RTW was analyzed using Kaplan Meier and Mann Whitney U (MWU), and multiple linear regression analysis was used to adjust for effect modification and confounding.

**Results:**

One hundred and eighty-two (182) patients were included with a median age of 59 years [IQR 54–62], including 95 women (52%). Patients who consulted an OMS were less often self-employed but did not differ on other patient and work-related characteristics. TKA patients who consulted an OMS returned to work later than those who did not (median 78 versus 62 days, MWU p < 0.01). The effect of consulting an OMS on time to RTW was modified by patients’ expectations in linear regression analysis (p = 0.05). A median decrease in time of 24 days was found in TKA patients with preoperative high expectations not consulting an OMS (p = 0.03), not in patients with low expectations.

**Conclusions:**

Consulting an OMS within 3 months after surgery did not result in a decrease in time to RTW in TKA patients. TKA patients with high expectations did RTW earlier without consulting an OMS. Intervention studies on how OMSs can positively influence a timely RTW, incorporating patients’ preoperative expectations, are needed.

**Supplementary Information:**

The online version contains supplementary material available at 10.1007/s10926-022-10068-1.

## Introduction

Worldwide there is a steep rising demand for total knee arthroplasty (TKA), especially among patients of working age. By 2030–2035 the majority of TKA patients in the US and UK will already be of working age [1–3]. Return to work (RTW) rates among these TKA patients vary between 40 and 98% with a mean time to return to work between 8 and 17 weeks [4, 5]. Time to RTW in TKA patients is often retrospectively measured and therefore prone to recall bias [5]. In the most recent prospective cohort study in the Netherlands only 24% of TKA patients returned to work completely at 3 months, which was 51% at 6 and 71% at 12 months [6]. These percentages are in contrast with orthopedic guidelines advising RTW within 3 months, starting gradually if needed [7]. Moreover patients who receive TKA have the greatest productivity and income loss when compared to other types of common surgery [8].

To decrease time to RTW among these TKA patients, attention should be paid to the beneficial and hindering factors for RTW within health care as well as occupational health. Known prognostic factors effecting RTW are patient characteristics, such as age, gender, Body Mass Index (BMI) and physical function score, as well as work-related characteristics, such as sense of urgency to RTW, having a handicap accessible workplace, preoperative sick leave, and having knee-straining work [4, 5]. Active referral by the orthopedic surgeon to an occupational health expert is expected to enhance RTW [9–12].

The occupational health experts in the Netherlands are the Occupational Medicine Specialists (OMS) who are physicians with four years post-graduate training in Occupational Medicine. Every employee has direct access to an OMS, on account of the employer. Self-employed patients can consult an OMS on their own account, though this consultation is not familiar to patients and health care professionals and thereby not frequently used.

By their specialized training the OMS has insight into the patient’s work demands in relation to the patient’s work ability. Subsequently the OMS can advise and support the RTW process, for instance by advising adjustment of working hours, working tasks (modified duties) or workplace adaptations using ergonomic principles. Patients who have limited access to these kinds of work adjustments and have difficulty performing work-related knee-straining activities may also be referred by their OMS to work rehabilitation [13]. Moreover, an OMS is able to discuss and stimulate the sense of urgency to RTW given the value of work in life [14]. Currently however, no evidence is available on whether time to RTW after TKA can be decreased by consulting an OMS. The aim of this study is to investigate whether TKA patients who consult an OMS within 3 months after surgery, return to work sooner than patients who do not consult an OMS.

## Methods

### Study Design and Population

A multi-center prospective cohort study among TKA patients was performed [12]. Patients were included from nine surgeons, working in seven hospitals, in five Dutch regions, to minimize selection bias. The hospitals varied from general hospitals, large teaching hospitals to tertiary university hospitals. Inclusion criteria were (1) patients undergoing TKA between June 2014 and March 2018, (2) aged 18 to 65 years (working age), (3) having a paid job, (4) self-reported intend to RTW after surgery and (5) provided information about consulting an OMS within 3 months after TKA or not. Data were collected before TKA and at 3, 6 and 12 months after surgery using a self-report questionnaire in Dutch. Patients could choose a paper or electronic version of the questionnaire to avoid selection bias based on patients’ computer literacy. At each measurement, non-responding patients were reminded up to two times after 2 weeks. Patients who were willing to participate but missed the preoperative measurement due to logistical reasons, were included in the follow-up measurements.

### Patient Characteristics

Patient characteristics were registered, such as date of birth, gender, body height and body mass. The latter two were used to calculate BMI. Patients were asked whether they had other diseases that were limiting their activities at work, with three categories: (1) No, (2) Yes, one disease that is limiting my activities at work, and (3) Yes, more than one disease that is limiting my activities at work. These categories were dichotomized into either ‘no’ (No) or ‘one or more other disease(s) that limits my activities at work’ (Yes), which was defined as comorbidity. Knee Injury and Osteoarthritis Outcome Score (KOOS) subscales on pain, symptoms and quality of live were filled out by the patients. All KOOS subscales are validated in Dutch and range from 0, representing extreme knee problems, to 100 representing no knee problems [15]. The Work Osteoarthritis or joint-Replacement Questionnaire (WORQ) was used for the work-related physical functioning score, and is also validated in Dutch [16]. The WORQ score consists of 13 items on work-related knee-straining activities, such as lifting and working with hands below knee-height. These 13 activities are assessed on a 5-point Likert scale, from 0 (extreme difficulty or unable to perform) to 4 (no difficulty at all), resulting in a converted total score between 0 (extreme difficulties) to 100 (no problems at all).

### Work-Related Characteristics

Work-related characteristics were also self-reported: being a bread winner (yes/no); being self-employed (yes/no); having a handicap accessible workplace (yes/no); and preoperative sick leave (yes/no). Patients’ preoperative expectation regarding work ability at 6 months after surgery was reported by using the single item work ability score (WAS). This score ranges from 0, at which score a patient expect no work ability at all at 6 months postoperative, to 10, an expected work ability as it was at lifetime best [17, 18]. Expected WAS was dichotomized with a cut-off point of 8 or higher defined as high expectations of postoperative work ability and lower than 8 as low expectations of postoperative work ability [17]. Having a knee-straining job was defined by patients who reported that they have to perform at least one of the following five activities ‘often’ or ‘always’: crouching, kneeling, clambering, taking the stairs or lifting [19, 20]. Resumed working hours at 6 and 12 months after surgery were self-reported and calculated as a percentage of self-reported regular working hours of each individual TKA patient. TKA patients reported their actual work ability on the single item Work Ability Score (WAS) again at 6 and 12 months. Furthermore, satisfaction with their physical work ability regarding the operated knee was reported on a single item score from 0, not satisfied at all to 10, totally satisfied.

### Occupational Medicine Specialist

Whether or not an OMS was consulted by TKA patients within 3 months after surgery was self-reported at the 3-month postoperative measurement.

#### Potential Confounders for RTW

Potential confounders for RTW were: age; gender; BMI; pain related to the knee (KOOS pain); symptoms related to the knee (KOOS symptoms); quality of life related to the knee (KOOS quality of life); perceived difficulty with work-related knee-straining activities (WORQ score); having a knee-straining job; preoperative sick leave; being self-employed; availability of a handicap accessible workplace and preoperative expected work ability (expected WAS) at 6 months postoperative [4, 5].

##### Study Size

A study size of 160 patients was deemed to be needed. This was based on an expected inclusion of six dichotomous or continuous variables in multiple linear regression analyses. Thereby we took into account that for every variable in the multiple analyses a minimum of 10 patients are required [21]. Finally, we assumed that 60 patients would be included without consult of an OMS and 100 patients with consult of an OMS after TKA.

### Statistics

Firstly, descriptive statistics were used to describe patient and work-related characteristics of TKA patients who did and did not consult an OMS. Differences between these groups were statistically tested at a significance level of p < 0.05.

Secondly, a Mann Whitney U non-parametric test (MWU) and a Kaplan Meier survival analysis were performed. The Kaplan Meier survival analysis was performed with the Wilcoxon (Breslow) test to give more weight to the first phase of RTW. This was done because of the importance of an early RTW and in line with the Dutch orthopedic guideline recommendation of RTW within 3 months.

Thirdly, to answer the question of whether or not consulting an OMS decreases (median) time to RTW after TKA, multiple linear regression analysis was performed in order to adjust for confounding and effect modification. To meet the assumptions of linear regression, the outcome measure, time to RTW, was transformed by taking the square root. Potential confounders were included in the multiple linear regression analysis that correlated with time to RTW at a significance level of p = 0.05 and showed a collinearity of < 0.7 using Spearman's Rank correlation coefficient with other potential confounders. This linear regression analysis was performed using the comprehensive method for association models [21]. First, OMS consultation was entered into the model. Initially, effect modifiers were identified because this meant that the presence of this factor differently affected (the square root of) time to RTW, if an OMS was consulted or not. A significance level of p < 0.10 was used to prevent potential relevant effect modifiers from being opted out. If effect modification was observed, a multiple linear regression analysis was performed within each stratum of the effect modifier to secure that the effect on RTW could be attributed to this specific factor. Subsequently, a confounder analysis was performed by adding one potential confounder at the time to the regression model, if needed per stratum of the effect modifier. The predictor variable with the largest effect on the regression coefficient of the OMS, and with an effect of at least a 10% change in the coefficient of the OMS, was then added to the model. These steps were repeated until none of the remaining potential confounders had an effect of at least a 10% change on the coefficient of the OMS, the number of cases was less than ten per variable or if no variables were left. Assumptions for applying linear regression analysis were checked on the final association model for homoscedasticity of errors, independency of errors using the Durbin-Watson test and normal distribution of errors.

Additionally, as a secondary outcome, working hours, experienced WAS and satisfaction with work ability at 6 and 12 months was assessed for differences between patients who did and did not consult an OMS.

All statistical analysis were performed using SPSS for Windows (Version 26.0; IBM Corp, Armonk, NY, USA).

## Results

### Patient and Work-Related Characteristics

**Fig. 1:**
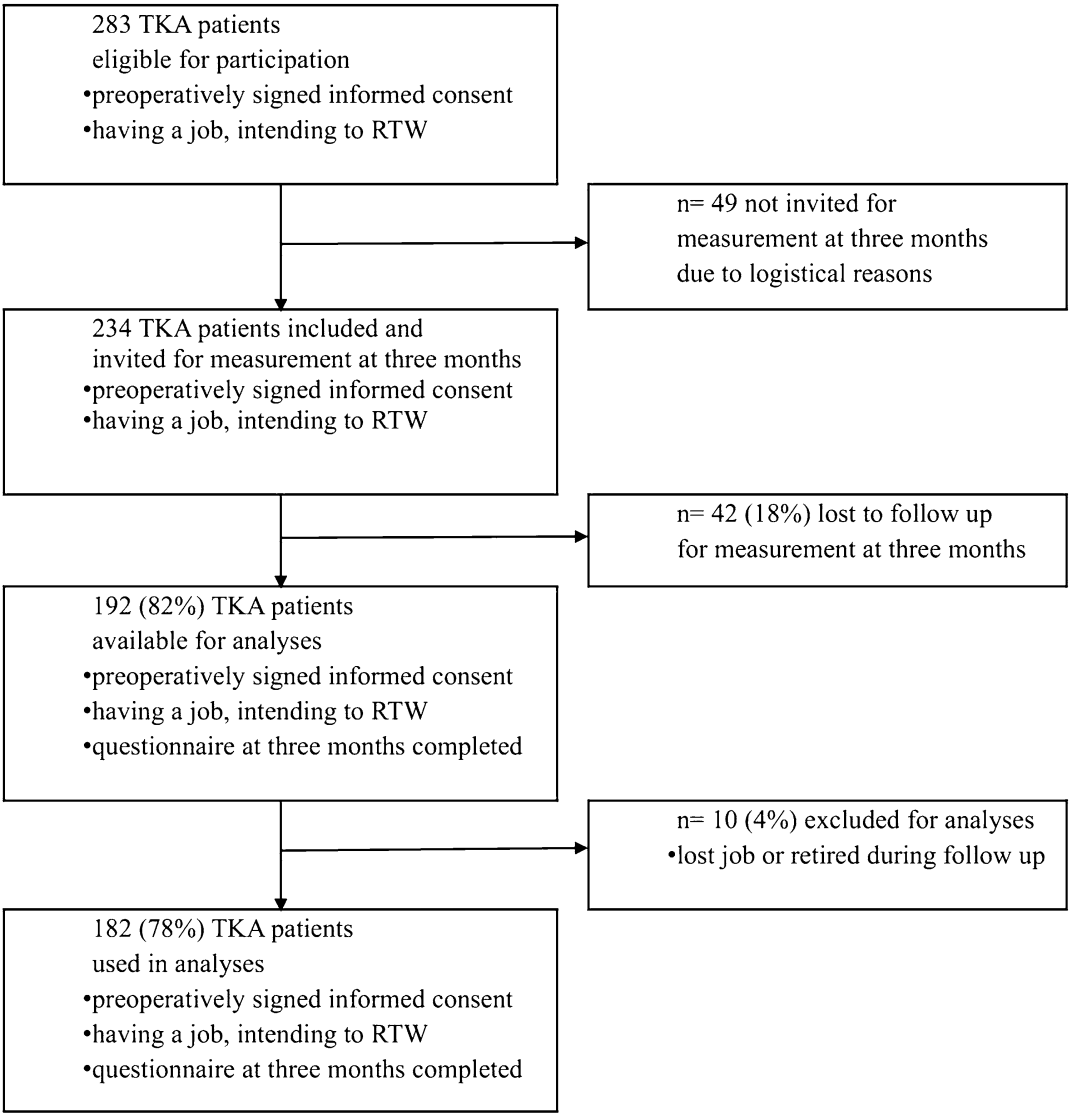
Inclusion flow chart of total knee arthroplasty patients who intended to return to work after surgery (*TKA* total knee arthroplasty, *RTW* return to work)

One hundred eighty-two (182) TKA patients were included (Fig. 1) with a median age at 3 months postoperative of 59 years [IQR 54–62], 87 men (48%), a median BMI of 29 [IQR 26–32], and a median KOOS symptoms score of 61 [IQR 46–71] (Table 1). Patients with and without an OMS consult did not differ regarding their personal and work-related characteristics such as comorbidity, KOOS symptoms and preoperative sick leave, except for being self-employed. Patients who consulted an OMS were less often self-employed (2%) than those not consulting an OMS (30%, p < 0.01). Preoperative KOOS subscales and WORQ scores were also not statistically different between TKA patients who did and did not consult an OMS.

**Table 1:** Patient and work-related characteristics at 3 months postoperative among all TKA patients and among patients who did and did not consult an occupational medicine specialist

Variable	All TKA patients		Consult of an occupational medicine specialist within 3 months postoperative				Test for differ-ence^a^
			NO		YES		
	Number (%)	Median [IQR]	Number (%)	Median [IQR]	Number (%)	Median [IQR]	p-value
	182		76 (41.8)		106 (58.2)		
Age		59 [54–62]		58 [53–61]		59 [55–62]	0.12
Gender							
Male	87 (48)		36 (47)		51 (48)		1.00
Female	95 (52)		40 (53)		55 (52)		
BMI		29 [26–32]		30 [27–34]		28 [26–31]	0.08
Comorbidity							
No	145 (80)		64 (84)		81 (76)		0.26
Yes	37 (20)		12 (16)		25 (24)		
KOOS symptoms, scale 0-100^b^		61 [46–71]		57 [43–71]		61 [46–71]	0.52
KOOS pain, scale 0-100^b^		67 [53–83]		67 [50–83]		67 [53–83]	0.84
KOOS quality of life, scale 0-100^b^		50 [38–63]		50 [38–63]		50 [44–63]	0.94
WORQ scale 0-100^b^		58 [46–73]		60 [42–75]		58 [48–73]	0.93
Breadwinner (yes)	114 (63)		42 (55)		72 (68)		0.10
Employment							
Employed	153 (84)		49 (65)		104 (98)		< 0.01
Self-employed	25 (14)		23 (30)		2 ( 2)		
Missing	4 ( 2)		4 ( 5)				
Knee-straining job (yes)	93 (51)		35 (46)		58 (55)		0.29
Handicap accessible workplace (yes)	143 (79)		60 (79)		83 (78)		0.85
Preoperative expected WAS scale 0-10^c^		8 [7, 8]		8 [7, 8]		8 [7, 8]	0.57
Missing^d^	32 (18)		14 (18)		18 (17)		
Preoperative full sick leave							
Yes	18 (10)		6 ( 8)		12 (11)		0.61
No	133 (73)		57 (75)		76 (72)		
Missing^d^	31 (17)		13 (18)		18 (17)		

*TKA* total knee arthroplasty, *IQR* inter quartile range, *BMI* body mass index, *KOOS* knee injury and osteoarthritis outcome score, *WORQ* Work Osteoarthritis or joint-Replacement Questionnaire, *WAS* Work Ability Score; due to the use of integers not every percentage add up to 100

^a^Fisher exact, Mann Whitney U when appropriate

^b^0 = extreme problems, 100 = no problems

^c^0 = no work ability at all, 10 = work ability as it was at lifetime best

^d^No preoperative measurement (n = 31, 17%)

### OMS Consultation and Effect on Time to Return to Work

**Fig. 2:**
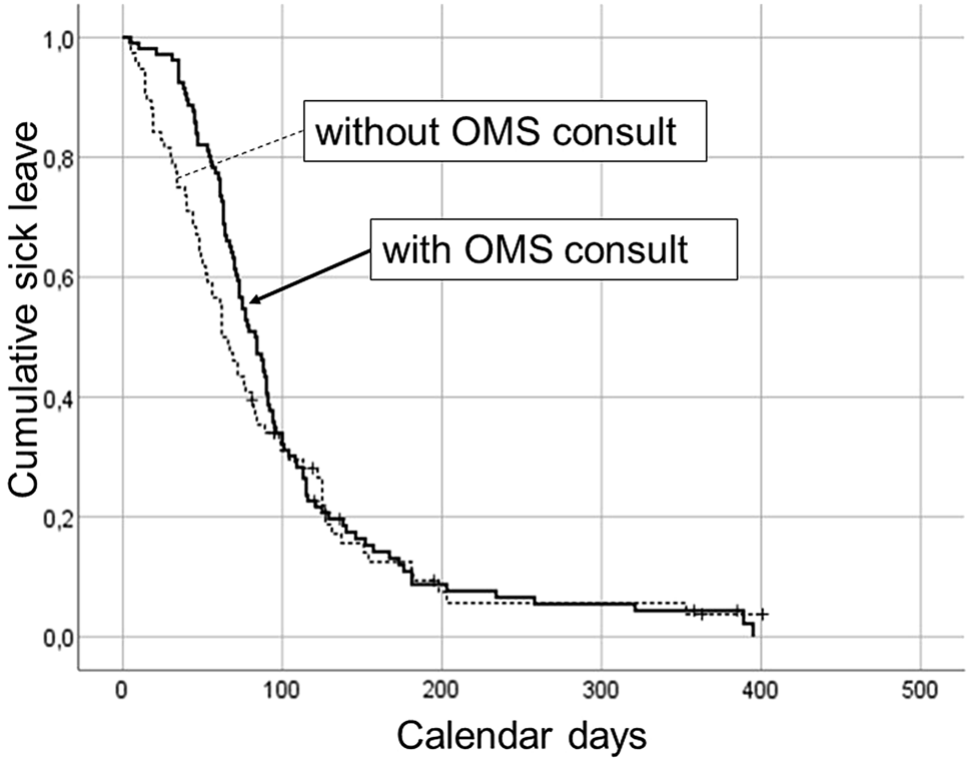
Kaplan Meier survival curves of time to RTW (calendar days) among TKA patients with (thick solid line) and without (dashed line) a consult of an occupational medicine specialist within 3 months postoperative (n = 182) differ, p = 0.03 (*RTW* return to work, *TKA* total knee arthroplasty, *OMS* occupational medicine specialist)

The Kaplan Meier survival curve of patients consulting an OMS (thick solid line) shows a later RTW compared to patients without consult of an OMS (dashed line) within 3 months postoperative (n = 182, p = 0.03 Fig. 2).

Patients, regardless whether or not they visited an OMS within 3 months postoperative, returned to work with a median of 72 days [IQR 47–108]. Patients who consulted an OMS returned to work later (median 78 [61–111]) than patients who did not consult an OMS (median 62 [34–102], MWU p < 0.01; Table 2).

**Table 2:** Days to return to work among TKA patients without and with consult of an occupational medicine specialist within 3 months postoperative

	Consult of an occupational medicine specialist within 3 months postoperative				Test for difference	
	No		Yes		Mann Whitney U	Adjusted regression coefficient OMS-consultation
	Days to RTW Median [IQR]	n	Days to RTW Median [IQR]	n	p-value	p-value
All working TKA patients	62 [34–102]	69	78 [61–111]	101	< 0.01	
Preoperative high expected WAS	46 [20–72]	40	70 [46–111]	57	< 0.01	0.03
Preoperative low expected WAS	106 [72–136]	16	86 [63–119]	26	0.46	0.69
Patients with paid employment	65 [40–115]	46	77 [60–108]	99	0.12	0.14
Patients being self-employed	46 [18–83]	23	Range 115–148	2	0.08	Uni-variable 0.04

*TKA* Total knee arthroplasty, *RTW* return to work, *IQR* inter quartile range; n, cases; *OMS* occupational medicine specialist, *WAS* work ability score

n = 170 patients because of 12 censored cases in Kaplan Meier analysis; n = 31 patients without preoperative measurement

The effect of consulting an OMS on time to RTW within 3 months postoperatively was modified by two variables in linear regression analysis, namely expected WAS (p = 0.054) and being self-employed (p = 0.062). Therefore, confounder analysis was performed in patients with high and low expected WAS and in patients with paid employment and being self-employed (Table 2). After controlling for confounding the following results were found. Only among patients who expected good postoperative work ability a significant association was found between the (square root of) days to RTW and a consult with an OMS when adjusted for having a knee- straining job and their KOOS pain level at 3 months postoperative (√days to RTW = √(7.875 + (1.404*OMS) + (2.524*Knee-straining job) + (− 0.027*KOOS-pain))). This association resulted in an R square of 0.274, meaning 27% of (the variation in) time needed to RTW was explained by (the variation in) a consult with an OMS, a knee straining job and pain level. TKA patients with high expected WAS returned to work 24 days later when an OMS was consulted compared to patients who did not consult an OMS (p = 0.03). TKA patients with low expected WAS returned to work at the same time whether an OMS was consulted or not (p = 0.69). TKA patients with paid employment returned to work at the same time with or without consulting an OMS (p = 0.14). Patients being self-employed seemed to return to work later when an OMS was consulted, however due to the low number of these patients (n = 2) this analysis could not be performed. Therefore, it could not be confirmed that employment was an effect modifier or confounder.

#### Working Hours and Work Ability

In patients with high expected WAS without consulting an OMS the number of working hours (median 32 [18–40]), experienced WAS (median 8 [IQR 7–8] and satisfaction with work ability (median 8 [IQR7-9]) at 6 months postoperative did not differ from patients with high expected WAS consulting an OMS (respectively 32 [IQR 16–40], 7 [IQR 7–8] and 8 [IQR 7–8]). The same was true at 12 months postoperative.

#### Preoperative Non-responders

Patients who did not respond to preoperative measurements did not differ significantly (at a p < 0.05 significance level) from patients who responded to preoperative measurements regarding patient and work-related characteristics (Supplementary data, Table 3).

## Discussion

Consulting an OMS did not show an earlier RTW among TKA patients. Moreover, in the group of TKA patients with high expectations an earlier RTW was seen in patients that had not consulted an OMS.

Regarding no earlier RTW in patients who consulted an OMS, four possible explanations can be given. First, it might be that OMSs advise a more conservative RTW trajectory than needed, to secure a safe recovery and sustainable RTW without increasing a risk of complications. At the moment no occupational health guideline regarding RTW advice for these patients is available in the Netherlands and other countries. The only guidance given is the practice-based recommendation in Dutch orthopedic TKA guideline stating that ‘RTW is possible within 3 months and should start gradually if needed [7]. Also the recently published Dutch and American physiotherapy guidelines, advice early and personalized progression of physical activity for TKA patients but lack recommendations regarding return to work [22, 23]. Recently the Dutch multidisciplinary practice guideline for occupational health professionals was developed for patients with low back pain and lumbosacral radicular syndrome [24]. Following this example a multidisciplinary occupational health guideline on prevention and work participation of knee osteoarthritis patients, as well as pre- and postoperative care in TKA patients can be of help for informed decision making and alleviate the burden of knee OA and TKA on patients, employers, health care and society [3, 8].

A second explanation might be that the timing of the consult with an OMS is not early enough to establish a decrease in time to RTW. In the Netherlands not every patient, for instance a self-employed patient, has free access to an OMS. If an employed patient can consult an OMS the waiting time for an appointment is often around 6 weeks which is when a consultation is mandatory to comply with the Dutch Gatekeeper law. This law states that a problem analysis regarding RTW has to be made by an OMS within the first 6 weeks of sick leave and this analysis should be used to make an RTW-plan by the employer and employee in the first 8 weeks of sick leave. However, managing the RTW-process for TKA patients should start earlier and preferably before surgery to have an effect on timely RTW after TKA. This is especially the case if hindering factors need to be managed like perceived difficulty with knee-straining activities, having a knee-straining job or a workplace that is not handicap accessible.

A third explanation might be that the previously mentioned waiting time for an OMS appointment can potentially lead to a wait-and-see attitude regarding RTW in patients who do not prefer or feel secure to RTW on their own accord. This would possibly be true in patients with less self-management skills, less confidence in their TKA recovery, less urgency to return to work or other reasons for a wait-and-see attitude regarding RTW. This possible wait-and-see attitude caused by the time to an OMS appointment is therefore also related to the fourth possible explanation, namely a possible selection bias.

This fourth possible explanation for no decrease in time to RTW among patients who consult an OMS might be selection bias based on psychosocial factors we did not measure, or so called reverse causation. In terms of the biopsychosocial model this study does not confirm selection bias based on biological factors, such as comorbidity, knee pain and symptoms (KOOS) or difficulty to perform work-related knee-straining activities (WORQ). However, selection bias based on psychosocial factors could be addressed more properly. To limit the number of questions, we prioritized prognostic variables for RTW regarding TKA patients described in literature. Remarkably, self-efficacy was not one of these variables. Patients that have less possibilities for personal job development or have less work recognition are recently found to have an increased time to RTW after TKA [25]. It seems plausible that these characteristics would be more present among patients consulting an OMS because of a possible need for psychosocial support.

### Patients Who Expect Good Postoperative Work Ability

A mixture of the aforementioned reasons 1, 2 and 3 can probably explain the later RTW in patients with high expected WAS that consulted an OMS compared to patients that did not consult an OMS. Regarding a probably conservative RTW trajectory by OMSs to secure a safe recovery and sustainable RTW, we could assess in our data that the early RTW in patients without consulting an OMS did not result in a worse outcome. The number of hours at work, the work ability of patients and the satisfaction with work ability did not differ between patients who did and did not consult an OMS.

Another explanation of an earlier RTW in patients with high expected WAS that did not consult an OMS might be a high self-efficacy which increases the probability of RTW on their own accord instead of waiting for an appointment with an OMS. Patients with high expected WAS are probably also patients with more possibilities for personal job development or more work recognition [8]. Moreover, expectations of work ability after surgery might (partly) be based on realistic insights in their physical ability in relation to physical job demands by these patients. If that is true then it can be argued that patients with high expected WAS would be able to safely RTW early and on their own accord in contrast to patients with low expected WAS given their worse physical ability in relation to their physical job demands. Qualitative findings also support the hypothesis that patients’ needs are partially based on having access to work adjustments and tools [13]. In line with these findings, patients in our study who have high expectations and do consult an OMS, might probably experience a lack of supportive interventions and for that reason consult an OMS.

### Future Directions

First of all, we would recommend to better inform OMSs regarding facilitating and hindering factors for RTW and corresponding median times for RTW regardless whether this is partial or full. This information might empower OMSs and thereby they can reassure their patients that resuming work in a timely manner has better prognosis for RTW [26]. As yet we do not know which exercise-based therapy and integrated care interventions are effective for soon, safe and sustainable RTW in TKA patients [27, 28]. Moreover, in line with the hierarchy of risk management it is more ethical to start with adjusting the work to the patient’s needs, especially if the work is knee-straining. Recent studies have shown that interventions supporting RTW in arthroplasty patients based on knowledge for safe recovery, also regarding work-related activities, and sustainable RTW are being developed [11, 29, 30]. Managing too high patient expectations has also been suggested in TKA patients for better patient-reported outcome as well as RTW [12, 31, 32]. The present study provides another perspective on the relevance of patients’ expectations, given that patients with high expectations who do not consult an OMS have a more timely RTW than their counterparts who consulted an OMS. OMSs might also pay attention to other patient needs than addressed by the potential confounders in our study because these only explained 24% of the later RTW in patients with high work ability expectations.

Independent of what advice or guidance supports an earlier RTW, it is also important to know whether this earlier RTW can be considered safe recovery and sustainable RTW. As said, TKA patients with high expectations without consulting an OMS returned to work earlier and had the same outcome of work ability at 6 and 12 months as their counterparts. We foresee, based on these findings, that an earlier RTW seems safe and sustainable, especially when patients have a high physical ability given their physical job demands and return to work on their own accord. Of course, more prospective studies are needed to confirm these findings.

### Promising Interventions

Hindering factors for RTW need to be addressed. Based on earlier research potential effective interventions might be work-directed rehabilitation in patients with a knee-straining job, facilitating arrangements to improve access to the workplace, preoperatively managing patient expectations regarding work ability after TKA and introducing ergonomic measures to decrease the physical workload [33, 34]. These measures could be initiated or coordinated by an OMS, even before surgery, so that the proposed interventions could be implemented in time [3, 35, 36]. Moreover, patients receiving a positive advice regarding RTW by their OMS as well as their orthopedic surgeon said that this was beneficial for their RTW [37]. When the orthopedic surgeon refers patients with hindering factors for RTW to their OMS, preferably before surgery, this might enhance a timely RTW if the OMS is able to act accordingly. RTW advise probably should also be individualized and needs involvement of the employer, as has been found in an intervention mapping approach to develop a clinical occupational advice intervention for knee arthroplasty patients [11, 38]. M/-eHealth could be another promising add-on intervention, since it can provide personalized and frequent advice for TKA patients regarding timely performance of activities based on for instance activity trackers, self-reported recovery and algorithms [29, 34]. Another possible effective element might be setting specific work activity goals in rehabilitation, since this resulted in an increased satisfaction with performing work-related activities in TKA patients [39]. Physiotherapists, especially those specialized in occupational health and ergonomics, could also add value because of their knowledge of the physical recovery of the patient and of assessing and (temporarily) adjusting physical job demands [33]. This dual approach of work directed care and corresponding adjustment of job demands might have potential to enhance (time to) RTW. Above all, given the lack of evidence, studies are needed that evaluate the effectiveness of physical rehabilitation for RTW, given that this care is received by most patients post-surgery [27].

### Strengths and Limitations

A strength of this study is its prospective multicenter design, in which seven hospitals throughout the Netherlands participated, resulting in a large number of working age TKA patients intending to RTW (n = 182). Another strength is that, as far as we know, this is the first study addressing the potential added value of consulting an OMS regarding RTW after TKA. Moreover, this study incorporated a priori chosen potential confounders for RTW although other confounders might still be present. And lastly, our multiple linear regression analysis, also focusing on modifiers and confounders of the effect of an OMS consult on RTW, can also be considered a strength of this study.

The most important limitation of our study is that selection bias is still possible due to the study design, being a prospective cohort study and not an intervention study. Another major limitation of our study is that we only had self-reported data and did not have information regarding the content and exact timing of the OMS consult. Also, the RTW process is complex and can be influenced by factors not measured, e.g. psychosocial factors such as support from a patients supervisor or colleagues at work [5]. Another limitation of the study is that it was originally designed as a cross-sectional preoperative measurement and pending approval for the postoperative measurements by the Medical Ethics Review Committee, the first consecutively 49 patients could not be invited for their 3-months measurement. Therefore, these patients were not eligible but we expect these to be random and not subject to whether or not an OMS was consulted within 3 months.

## Conclusion

Consulting an OMS within 3 months after surgery did not result in an earlier RTW in TKA patients. TKA patients with preoperative high expectations of work ability did even RTW earlier if they had not consulted an OMS compared to their counterparts. Given the increasing number of working age TKA patients worldwide, these findings strengthen the plea for more research on interventions to decrease time to RTW, incorporating the positive effects of high expectations on RTW.

## Supplementary Information

Below is the link to the electronic supplementary material.

Supplementary file1 (DOCX 35 kb)

## Data Availability

The datasets generated during and/or analysed during the current study are available from the corresponding author on reasonable request.
