# Supplementary material for: Does Consulting an Occupational Medicine Specialist Decrease Time to Return to Work Among Total Knee Arthroplasty Patients? A 12-Month Prospective Multicenter Cohort Study
Source: J Occup Rehabil. 2022 Sep 9;33(2):267–76. doi: 10.1007/s10926-022-10068-1 (PMC10172284; doi:10.1007/s10926-022-10068-1)
Supplement: Supplementary file 1 — Supplementary file1 (DOCX 35 kb) [file 10926_2022_10068_MOESM1_ESM.docx]

Supplementary data

Table 3 Patient and work-related characteristics at three months postoperative among all TKA patients and among patients who did and did not respond to preoperative measurement

| Variable | All TKA patients | | Preoperative measurement | | | | Test for differ-ence^a^ |
| --- | --- | --- | --- | --- | --- | --- | --- |
|  |  |  | NO | | YES | |  |
|  | number (%) | median [IQR] | number (%) | median [IQR] | number (%) | median [IQR] | p-value |
|  | 182 |  | 31 (17) |  | 151 (83) |  |  |
| Age |  | 59 [54-62] |  | 61 [55-63] |  | 58 [54-62] | 0.09 |
| Gender |  |  |  |  |  |  |  |
| Male | 87 (48) |  | 18 (58) |  | 69 (46) |  | 0.15 |
| Female | 95 (52) |  | 13 (42) |  | 82 (54) |  |  |
| BMI |  | 29 [26-32] |  | 29 [26-31] |  | 29 [26-32] | 0.62 |
| Comorbidity |  |  |  |  |  |  |  |
| No | 145 (80) |  | 28 (90) |  | 117 (78) |  | 0.08 |
| Yes | 37 (20) |  | 3 (10) |  | 34 (23) |  |  |
| KOOS symptoms,  scale 0-100^b^ |  | 61 [46-71] |  | 64 [50-75] |  | 57 [46-71] | 0.18 |
| KOOS pain,  scale 0-100^b^ |  | 67 [53-83] |  | 75 [58-92] |  | 67 [50-81] | 0.07 |
| KOOS quality of life, scale 0-100^b^ |  | 50 [38-63] |  | 56 [44-69] |  | 50 [38-63] | 0.17 |
| WORQ  scale 0-100^b^ |  | 58 [46-73] |  | 62 [44-77] |  | 58 [48-73] | 0.50 |
| Breadwinner (yes) | 114 (63) |  | 24 (77) |  | 90 (60) |  | 0.20 |
| Employment |  |  |  |  |  |  |  |
| Employed | 153 (84) |  | 27 (87) |  | 126 (83) |  | 0.29 |
| Self-employed | 25 (14) |  | 3 (10) |  | 22 (15) |  |  |
| Missing | 4 ( 2) |  | 1 ( 3) |  | 3 ( 2) |  |  |
| Knee-straining job (yes) | 93 (51) |  | 15 (48) |  | 78 (52) |  | 0.29 |
| Handicap accessible workplace (yes) | 143 (79) |  | 23 (74) |  | 120 (80) |  | 0.85 |
| Preoperative expected WAS scale 0-10^c^  Missing^d^ | 32 (18) | 8 [ 7- 8] | n.a.  31 (100) |  | 1 (1) | 8 [ 7- 8] | n.a. |
| Preoperative full sick leave |  |  |  |  |  |  |  |
| Yes | 18 (10) |  |  |  | 18 (12) |  | n.a |
| No | 133 (73) |  |  |  | 133 (88) |  |  |
| Missing^d^ | 31 (17) |  | 31 (100) |  |  |  |  |

^a^Fisher exact, Mann Whitney U when appropriate; ^b^0=extreme problems, 100=no problems; ^c^0=no work ability at all, 10=work ability as it was at lifetime best; ^d^No preoperative measurement (n=31, 17%); TKA, total knee arthroplasty; IQR, inter quartile range; BMI, body mass index; KOOS, knee injury and osteoarthritis outcome score; WORQ, Work Osteoarthritis or joint-Replacement Questionnaire; WAS, Work Ability Score; n.a., not applicable; due to the use of integers not every percentage add up to 100.
